# Perceived safety in digital information environments: objective violence, online disinformation, internet use, and victimization across Europe, 2002–2023

**DOI:** 10.3389/fpsyg.2026.1918767

**Published:** 2026-08-26

**Authors:** Xinyun Tang, Guoliang Lu

**Affiliations:** 1 School of Journalism and Communication, Shanghai International Studies University, Shanghai, China; 2 Institute of Ecological Sociology, Fujian Academy of Social Sciences, Fuzhou, China; 3 School of Journalism and Communication, Chongqing University, Chongqing, China

**Keywords:** disinformation, European Social Survey, homicide, internet use, media psychology, perceived safety, risk communication, victimization

## Abstract

**Introduction:**

Perceived safety is a psychologically consequential judgment shaped by objective threats, prior victimization, and mediated information. However, few studies jointly examine longitudinal crime data, national digital-information environments, and individual Internet use.

**Methods:**

We integrated European Social Survey Rounds 1–11 with homicide data from the United Nations Office on Drugs and Crime, Digital Society Project indicators, and World Development Indicators. Study 1 examined 308 country–round–year cells from 38 European countries, representing 442,100 respondents, using weighted country and survey-round fixed-effects models. Study 2 analyzed 408,149 respondents from 37 countries and 345 country–round–year cells using cell fixed-effects models.

**Results:**

In Study 1, conventional country-clustered models indicated that within-country increases in lagged homicide were associated with lower perceived safety (−1.42 percentage points per within-country SD, 95% CI [−2.47, −0.38], *p* = 0.008), whereas increases in expert-coded government online disinformation were associated with higher perceived safety (+0.87 points, 95% CI [0.34, 1.40], *p* = 0.001), particularly in countries with higher long-run homicide rates (+0.52-point interaction, 95% CI [0.24, 0.80], *p* < 0.001). However, the corresponding restricted wild-cluster bootstrap *p*-values were 0.087, 0.052, and 0.091, and country-specific trends attenuated the two main effects. In Study 2, more frequent Internet use was associated with higher perceived safety (+1.31 points per SD, 95% CI [0.83, 1.78], *p* < 0.001). Victimization was associated with substantially lower perceived safety (−13.65 points), but the positive association between Internet use and perceived safety was stronger among victims (+2.67-point interaction, 95% CI [2.00, 3.34], *p* < 0.001). These three individual-level terms remained significant at *p* < 0.001 under 9,999-replication wild-cluster inference, and their directions remained unchanged when each cell received equal total weight.

**Discussion:**

The national-level findings should be considered provisional. The individual-level findings are consistent with—but do not establish—mechanisms involving access to information, social support, or perceived efficacy. Given the observational design, all results represent conditional associations rather than causal effects.

## 1 Introduction

Feeling safe while moving through one’s local environment is a basic psychological condition of everyday autonomy. Persistent unsafety can constrain mobility, reduce participation in work and community life, weaken informal social interaction, and contribute to poorer functioning and wellbeing (Ejrnæs and Scherg, 2022; Hart et al., 2022; Kaiser et al., 2025). Recent longitudinal evidence further indicates that victimization-related changes in fear and unsafety can vary with neighborhood disadvantage, underscoring that perceived safety is embedded in both individual experience and local conditions (Kaiser et al., 2025). Perceived safety is therefore more than a descriptive opinion about crime. It is a situated judgment with implications for behavior, wellbeing, and access to public life.

Conceptual precision is important because the literature often uses fear of crime, perceived risk, worry, and perceived safety interchangeably. Fear is usually treated as an affective response, perceived risk as a cognitive estimate of victimization likelihood, and perceived safety as a broader appraisal of whether a setting can be navigated without unacceptable threat. Recent systematic reviews distinguish these constructs and show that inconsistent definitions and single-item measures contribute to heterogeneous findings across studies (Hart et al., 2022; Spielvogel et al., 2024). The European Social Survey (ESS) item asking how safe respondents feel walking alone in their local area after dark does not constitute a multidimensional fear-of-crime scale. It nevertheless captures a consequential appraisal of vulnerability that combines anticipated threat, environmental confidence, and likely behavioral freedom, and it remains widely used in recent comparative European research (Ejrnæs and Scherg, 2022; Nazzari et al., 2026). We therefore refer to the outcome as perceived safety rather than emotional fear.

Perceived safety is not a direct readout of recorded crime. Objective violence matters, but its psychological meaning is filtered through prior experience, social position, neighborhood cues, institutional confidence, and communication. Studies that distinguish perceived unsafety from emotional fear show that violent and property crime, neighborhood conditions, and prior appraisals can have different relationships with each component (Camacho Doyle et al., 2022). Recent cross-national research likewise identifies victimization, gender, age, welfare institutions, inequality, and macroeconomic conditions as important correlates, while objective crime indicators explain only part of the variation (Visser et al., 2013; Ejrnæs and Scherg, 2022; Nazzari et al., 2026). This imperfect correspondence is not simply measurement error. It reflects the fact that citizens rarely observe population risk directly and must infer it from lived experience and socially circulated information.

The social amplification of risk framework treats media, institutions, and interpersonal networks as amplification stations that transform signals about hazards (Kasperson et al., 1988; Kasperson et al., 2022). A signal may be amplified through repetition, vivid imagery, moral framing, uncertainty, or perceived uncontrollability; it may be attenuated through normalization, attention displacement, reassurance, or restricted visibility. Current risk-information-seeking research similarly treats information insufficiency as a condition that motivates communication use. Meta-analytic evidence identifies information sufficiency, current knowledge, and especially social norms as predictors of seeking, with stronger relations when risk is personally relevant (Liu et al., 2022). Together, these perspectives imply that communication can either magnify threat or increase control. The net psychological association depends on what information is available, which sources are used, and whether users can translate information into effective action.

Crime communication illustrates this duality. Recent evidence links sensational local crime-news formats with heightened fear (Shin and Watson, 2024), but a scoping review of 72 studies found a predominance of null associations and substantial heterogeneity across exposure and outcome measures (Spielvogel et al., 2024). Yet source, content, audience, and social position matter. Comparative work distinguishes traditional news, social media, and alternative information sources, and finds that their associations with fear of violence are not interchangeable (Näsi et al., 2021). Ideology and source selection can alter how crime information is interpreted (Andersson, 2025), while intersectional analyses indicate that media–fear relationships vary across demographic groups rather than operating as universal main effects (Rosenberger et al., 2023). These findings make broad claims about “media exposure” theoretically insufficient.

Digital communication increases both informational reach and heterogeneity. Social media can circulate decontextualized incidents faster than official statistics can correct impressions, and posts about crime tend to overrepresent violent or sexual offenses relative to their prevalence (Prieto Curiel et al., 2020). Crime-specific digital exposure may consequently heighten concern: social-media consumption has been associated with fear of crime among young adults (Intravia et al., 2017), and recent evidence indicates that users of police-scanner social-media groups report greater fear of violent crime (Kim et al., 2026). General Internet use, however, is a different exposure. It may include work, interpersonal communication, navigation, local alerts, service access, entertainment, and information seeking. These affordances can reduce uncertainty, maintain social connection, and increase perceived capacity to respond. A frequency measure of general Internet use therefore captures digital embeddedness, not a dose of threatening crime content.

Empirical evidence on general Internet use and safety perception is correspondingly mixed. A Chinese cross-sectional study found that Internet use and use frequency were associated with lower perceived social safety (Wang et al., 2022). In contrast, Ding et al. (2026) analyzed 33,181 respondents in ESS 2023 and reported a modest positive association between digital-technology use intensity and neighborhood safety perception; the association was stronger among respondents with victimization experience. The latter study interpreted digital technology as a possible source of information, social support, and efficacy, but also emphasized that its cross-sectional design could not establish temporal ordering and lacked objective crime statistics. It explicitly identified longitudinal comparative analysis and integration of structural safety conditions as priorities. The present study addresses those two limitations while retaining a conservative interpretation of general Internet-use frequency.

A second digital layer is the national information environment. Disinformation is not only inaccurate content encountered by individuals; it can also be an organized political communication practice embedded in media systems (Bennett and Livingston, 2018; Freelon and Wells, 2020). Governments may use social media to disseminate misleading narratives, attack independent sources, redirect attention, or cultivate favorable impressions of performance. Research on informational autocracy emphasizes that contemporary political control may rely on persuasion, selective visibility, and reputation management as much as overt repression (Guriev and Treisman, 2019). Cross-national work further suggests that resilience to online disinformation depends on political polarization, media institutions, and public-service information capacity (Humprecht et al., 2020). Such environments may increase uncertainty and distrust, but they may also suppress the salience of negative conditions. Misinformation psychology therefore provides no unambiguous directional prediction for neighborhood safety: belief, source evaluation, prior knowledge, and exposure all condition downstream judgments (Ecker et al., 2022).

The national and individual digital layers must be kept analytically distinct. Expert-coded government online disinformation is a contextual property of a political communication system. It does not show that a particular ESS respondent encountered or believed a crime-related falsehood. Conversely, individual Internet-use frequency does not indicate whether the respondent used social media, local news, messaging, navigation, or public services. Treating either measure as direct exposure to threatening content would create a construct-validity error. Their joint analysis is still useful because it separates two questions: whether population safety changes alongside the surrounding information environment, and whether people who are more digitally connected differ from otherwise similar people within the same survey context.

Recent person-specific media-effects research shows that digital-media associations can differ substantially across individuals and that aggregate average effects may obscure positive, negative, and near-zero responses (Valkenburg et al., 2021). Direct or household victimization is a particularly relevant susceptibility factor. Recent longitudinal research indicates that victimization can reduce safety and increase fear, with effects varying by prior victimization and neighborhood conditions (Ejrnæs and Scherg, 2022; Kaiser et al., 2025). It may heighten vigilance to alarming content, but it may also increase the marginal value of location-specific information, rapid contact, navigation, and formal or informal support. The direction of moderation is therefore not self-evident. The recent ESS finding of a stronger positive digital-use association among victims provides a theoretically important pattern that warrants replication across countries and survey rounds (Ding et al., 2026).

The present research integrates these arguments in two complementary observational studies. Study 1 constructs a repeated country–round–year panel from ESS Rounds 1–11 and links population safety perceptions to lagged intentional homicide and expert-coded government online disinformation. Its within-between specification distinguishes short-term change within a country from stable cross-national differences. Study 2 compares respondents within the same country, ESS round, and fieldwork year, thereby holding the shared national and survey context constant while estimating associations of Internet-use frequency and victimization. This design does not turn observational data into causal evidence, but it reduces several common ambiguities by separating objective risk, national information context, individual media behavior, and direct experience.

H1 predicts that within-country increases in objective homicide will be associated with lower perceived safety. Because government online disinformation could either amplify uncertainty or attenuate the visibility of unfavorable conditions, RQ1 asks whether within-country changes in government online disinformation are associated with perceived safety, and RQ2 asks whether that association varies by a country’s long-run homicide context. H2 predicts that, within the same country–round–year context, more frequent Internet use will be associated with higher perceived safety. H3 predicts that this positive association will be stronger among respondents reporting personal or household victimization. Exploratory analyses assess whether national homicide and disinformation environments condition the individual Internet-use association.

## 2 Materials and methods

### 2.1 Design overview and data sources

We conducted secondary analyses of anonymized public-use ESS data and linked them to independently produced annual country indicators. The research comprises two connected observational studies with different units of analysis. Study 1 uses repeated cross-sectional national aggregates to estimate whether changes in country conditions are accompanied by changes in population safety perceptions. Study 2 retains respondents and absorbs country–round–year cells, estimating whether individuals who differ in Internet use and victimization also differ in safety while sharing the same national survey context. Neither study follows the same individuals over time, and neither identifies a causal media effect.

ESS began in 2002 and uses probability-based national samples, standardized questionnaires, translation protocols, and harmonized fieldwork procedures (European Social Survey European Research Infrastructure, 2024). We used Rounds 1–11, including interviews conducted from 2002 through 2023. The integrated source file contained 518,597 respondents in 433 country–round–year cells and 39 countries before analysis-specific restrictions. Country and fieldwork year were harmonized to ISO3 and calendar-year identifiers. Descriptive statistics and cell aggregates used the ESS post-stratification weight (pspwght), which already incorporates the design-weight adjustment; it was not multiplied by dweight. In Study 2, pspwght was rescaled to a mean of 1 within each country–round–year cell. This rescaling preserves relative survey adjustments within cells but leaves total cell contribution proportional to the number of analyzed respondents. We therefore also estimated a sensitivity specification in which every cell received equal total weight.

### 2.2 Outcome definition and inferential scope

The outcome was the ESS item aesfdrk: “How safe do you—or would you—feel walking alone in this area after dark?” Responses were very safe, safe, unsafe, and very unsafe. The primary outcome classified very safe/safe as 1 and unsafe/very unsafe as 0. The national analysis used the weighted proportion coded 1, and the respondent analysis used the binary response. Robustness analyses retained the four-category ordering, recoded so higher scores indicated greater safety. This binary operationalization facilitates percentage-point interpretation and corresponds to the substantive distinction between feeling broadly safe and unsafe; the ordinal analysis tests whether conclusions depend on that threshold.

We interpret this item as perceived safety, not as a complete measure of emotional fear of crime. It is anchored in a local nighttime scenario, but respondents may answer from direct experience, anticipated vulnerability, neighborhood cues, or a generalized sense of security. The wording also permits a hypothetical response from people who rarely walk alone after dark. Accordingly, the models estimate associations with a situated appraisal and should not be generalized to all components of fear, worry, perceived victimization probability, or avoidance behavior.

### 2.3 Study 1: national repeated cross-sectional panel

Study 1 aggregated respondents to country–round–year cells. Cells required at least 200 nonmissing safety responses, complete macro covariates, and at least four Digital Society Project (DSP) coders in each year contributing to the exposure window (Digital Society Project, 2026). The final panel comprised 308 cells in 38 countries and represented 442,100 respondents with valid safety responses. Cell safety and victimization prevalence were calculated with ESS weights. The minimum-size rule reduced instability in small national subsamples; a threshold of 500 was examined as a robustness check.

Objective violence was measured with intentional homicide per 100,000 population from the World Development Indicators (World Bank, 2026), whose metadata identify the United Nations Office on Drugs and Crime as the underlying source. Homicide was selected because it is more comparable across countries than recorded nonlethal offenses, although it remains an imperfect proxy for the local environment evaluated by respondents. We averaged the first and second calendar years before fieldwork, applied log(1 + rate), and decomposed the result into the country mean and the deviation from that mean. The lag window reduces simultaneity and allows time for objective conditions to enter public awareness.

Government online disinformation was measured with DSP indicator v2smgovdom, which asks experts how often governments and their agents use social media to disseminate misleading viewpoints or false information to influence the domestic population. We reversed the original orientation so higher values represented more disinformation, averaged the two pre-fieldwork years, and retained the associated coder-count information for quality restrictions. Like homicide, the exposure was separated into the country mean and within-country deviation. Party disinformation, online pluralism, and other coding-quality thresholds were used only in robustness analyses. The DSP measure is political and system-level; it is not a respondent-level measure of exposure, belief, or crime-specific content.

Macro covariates were log real GDP per capita, unemployment, urban population share, and the percentage of individuals using the Internet, obtained from the World Development Indicators (World Bank, 2026) and aligned to fieldwork year. These variables address broad development, labor-market, settlement, and diffusion differences that may co-evolve with safety perceptions and information environments. A robustness model additionally adjusted for World Governance Indicators rule of law. Cell victimization prevalence was excluded from the initial specifications because it is partly downstream of crime conditions and was unavailable for some substantive interpretations; it was added in Model 4 to assess whether direct household experience accounted for the focal associations.

### 2.4 Study 1 statistical analysis

Study 1 used weighted least squares, weighting cells by the number of valid safety responses. Primary models included country fixed effects and ESS-round fixed effects. Country fixed effects remove stable national characteristics, including long-run cultural response tendencies and time-invariant media-system differences. Round fixed effects absorb Europe-wide survey-period shocks and common questionnaire-era changes. To assess temporal confounding, additional models replaced round fixed effects with calendar-year fixed effects, included both sets of period indicators, and added country-specific linear time trends to the round- and calendar-year specifications. Standard errors were clustered by country. Coefficients were multiplied by 100 and are reported as percentage-point differences in the proportion feeling safe.

The within-between decomposition represented each time-varying focal variable as a country mean and a deviation from that mean (Bell and Jones, 2015). H1 concerns the standardized within-country homicide component. RQ1 concerns the standardized within-country disinformation component. To examine conditional context, Model 3 included long-run homicide × within-country disinformation, within-country homicide × long-run disinformation, and within-country homicide × within-country disinformation. The first interaction asks whether short-term changes in the information environment have different associations in countries that have historically experienced different homicide levels; it does not imply that information manipulation changes objective violence.

Models were estimated sequentially: objective homicide alone (Model 1), homicide plus disinformation (Model 2), the contextual interaction specification (Model 3), and the addition of cell victimization prevalence (Model 4). Sensitivity analyses altered cell weights, minimum sample size, DSP coder threshold, lag window, outcome scale, pandemic-round inclusion, governance controls, the disinformation indicator, and temporal fixed-effects structure. Leave-one-country-out models assessed whether a single national series determined the results. Because 38 clusters may yield optimistic asymptotic *p-*values, focal Model 3 terms were evaluated with 9,999-replication restricted wild-cluster bootstrap-t tests using Rademacher weights (MacKinnon et al., 2023). We report both conventional and small-cluster inference rather than selecting the more favorable result.

### 2.5 Study 2: individual internet use and victimization

Study 2 used ESS rounds containing a harmonizable Internet-frequency measure: Rounds 1–5 and 8–11. Rounds 6–7 lacked a comparable item and were excluded. The final sample contained 408,149 respondents in 345 country–round–year cells and 37 countries after complete-case restrictions for the outcome and focal variables. The old item netuse ranged from no access/never to daily use; the later item netusoft ranged from never to every day. Each was rescaled to 0–1 and then standardized within country–round–year cells using normalized survey weights. Within-cell standardization means the focal coefficient represents relative Internet-use frequency among people surveyed in the same national-period context, not cross-national Internet penetration or technological development.

Victimization was measured by crmvct, which asks whether the respondent or a household member had been the victim of burglary or assault during the previous 5 years. Yes was coded 1 and no 0. This item identifies direct household relevance but combines offense types, respondents and household members, and a broad time window. Individual covariates were female gender, age and age squared, years of education plus a missingness indicator, and unemployment. A reduced-sample robustness model added household-income decile. These controls were selected before model estimation because they are established correlates of Internet use, victimization exposure, and safety appraisal.

### 2.6 Study 2 statistical analysis

We estimated weighted linear probability models after weighted within-cell demeaning of the outcome and all respondent-level predictors, which is algebraically equivalent to including country–round–year fixed effects for the focal slopes. In the primary specification, pspwght was normalized to a mean of 1 within cells. The resulting estimand is a sample-size-weighted average of within-cell associations: cells with more analyzed respondents contribute more total weight. A sensitivity model instead normalized weights to the same total within every cell, estimating an equal-cell average association. Country-clustered standard errors were used in the primary model to allow arbitrary dependence across respondents and survey cells within countries. Model I1 estimated the Internet-use association and victimization difference. Model I2 added Internet use × victimization. The Internet coefficient in Model I2 is the slope among nonvictims; the victim slope is the sum of the Internet main effect and interaction.

Predicted probabilities were calculated over Internet use from −2 to +2 within-cell SD for victims and nonvictims, averaging over the observed distribution of covariates and cells. Robustness analyses used the ordinal outcome, separate old and new Internet measurement eras, equal respondent weights, equal total weight per country–round–year cell, clustering by country–round–year cell, exclusion of ESS Round 10, and household-income adjustment. Because the primary respondent models clustered within only 37 countries, the Internet-use, victimization, and Internet use × victimization coefficients in Model I2 were additionally evaluated with 9,999-replication restricted wild-cluster bootstrap-t tests using Rademacher weights. Exploratory models interacted Internet use with standardized national government disinformation and lagged homicide. Because national context is constant within each country–round–year cell, its main effect is absorbed and only variation in the individual Internet-use slope is identified. These cross-level tests were not preregistered and are treated as hypothesis-generating.

### 2.7 Software, transparency, and reporting

Analyses were conducted in Python 3.13.5 using pandas 2.2.3, NumPy 2.3.5, SciPy 1.17.0, statsmodels 0.14.6, patsy, and matplotlib 3.10.8. Two-sided *p* values and 95% confidence intervals are reported. No *post hoc* variable search was used to replace the prespecified primary models, and the national results are presented with both conventional country-clustered and restricted wild-cluster inference.

The public replication package contains the exact analysis script, non-disclosive national and country–survey-cell aggregates, machine-readable model outputs, figure files, data dictionaries, and a source-download manifest. ESS respondent-level microdata are not redistributed because they remain subject to ESS access terms. Full reproduction requires free registration and downloading the relevant ESS round files, followed by execution of the supplied code with the public macro data. Reporting follows STROBE principles for observational studies, supplemented by explicit statements on level of analysis, temporal alignment, weighting, clustering, and the noncausal scope of inference.

## 3 Results

### 3.1 Samples and descriptive patterns

Table 1 summarizes the two analytical samples. Study 1 included 308 country–round–year cells in 38 countries, with a median of multiple observations per country across ESS Rounds 1–11 and 442,100 valid safety responses represented. Across cells, the weighted mean proportion feeling safe was 78.82% (SD = 10.41 percentage points; range = 41.04–95.25%). Mean victimization prevalence was 16.18%, and the mean two-year lagged homicide rate was 1.76 per 100,000, with substantial right skew before transformation. The mean share of Internet users was 72.92%, reflecting the long 2002–2023 observation period.

**Table 1:** Study samples and descriptive statistics.

Analysis	Contextual units	Respondents	Selected descriptives
National panel	38 countries; 308 country–round–year cells	442,100 respondents represented	Weighted safety = 78.82%; victimization prevalence = 16.18%; mean homicide = 1.76 per 100,000
Individual Internet study	37 countries; 345 country–round–year cells	408,149 respondents	Weighted safety = 77.69%; victimization = 16.11%; Internet use (0–1) = 0.616; mean age = 46.41

National statistics are across analysis cells unless otherwise stated. Individual statistics use the ESS post-stratification weight (pspwght), which already incorporates the design-weight adjustment and was not multiplied by dweight. In the primary respondent analysis, pspwght was normalized to a mean of 1 within each country–round–year cell. The full sample flow and variable descriptives appear in Supplementary Tables S1, S4, S9.

Study 2 included 408,149 respondents in 345 country–round–year cells and 37 countries. Weighted estimates indicated that 77.69% felt safe and 16.11% reported personal or household victimization. Mean rescaled Internet-use frequency was 0.616 (SD = 0.431 before within-cell standardization). Respondents had a weighted mean age of 46.41 years, 51.68% were female, and mean education was 12.36 years. The sample spans both the early Internet era measured in Rounds 1–5 and the later high-diffusion era measured in Rounds 8–11.

### 3.2 Study 1: objective violence and the national digital-information environment

**Figure 1:**
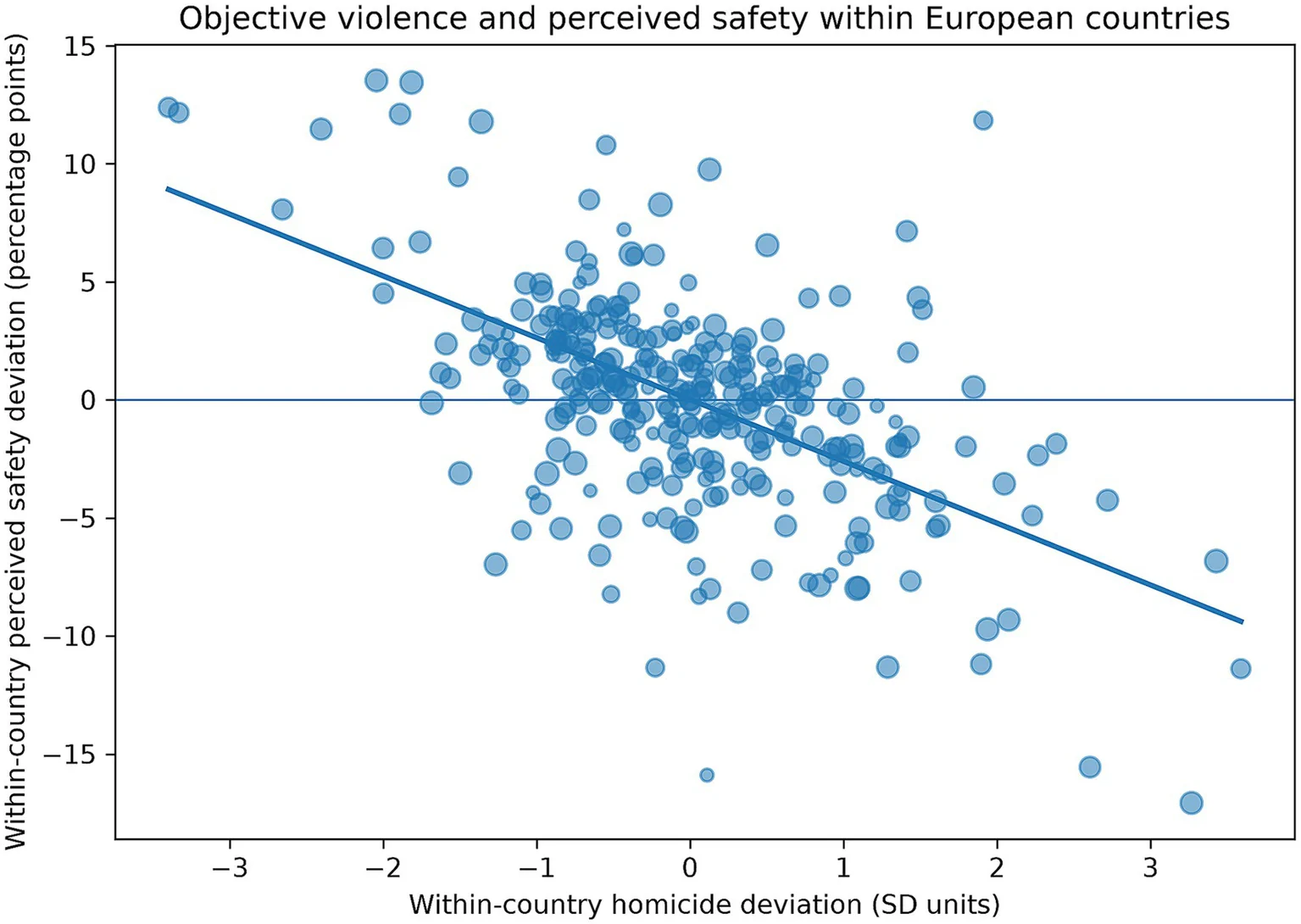
Objective violence and perceived safety within European countries. Point size is proportional to the square root of the number of respondents in the country–round–year cell. The displayed line is descriptive; inferential estimates are from the adjusted fixed-effects models. Alt text: Scatterplot of within-country standardized homicide deviations against within-country deviations in the percentage feeling safe. The fitted line slopes downward.

H1 received suggestive rather than definitive support. In the conventional country-clustered Model 1, a one-SD increase in lagged log homicide relative to a country’s own long-run level corresponded to a 1.61-percentage-point reduction in perceived safety (95% CI [−2.78, −0.45], *p* = 0.007). Adding government disinformation in Model 2 produced a similar coefficient (−1.64 points, *p* = 0.006). In Model 3, the conventional estimate was −1.42 points (95% CI [−2.47, −0.38], *p* = 0.008) (Table 2 and Figure 1). This direction is consistent with population safety declining when lethal violence rises relative to a country’s history, but the small-cluster and country-trend results reported below preclude treating H1 as firmly established.

**Table 2:** National perceived-safety models.

Model	Term	b (pp)	95% CI	*p*
M1 objective risk	Within-country lagged log homicide	−1.61	[−2.78, −0.45]	= 0.007
M2 add disinformation	Within-country lagged log homicide	−1.64	[−2.81, −0.47]	= 0.006
M2 add disinformation	Within-country government online disinformation	0.80	[−0.04, 1.64]	= 0.062
M3 contextual amplification	Within-country lagged log homicide	−1.42	[−2.47, −0.38]	= 0.008
M3 contextual amplification	Within-country government online disinformation	0.87	[0.34, 1.40]	= 0.001
M3 contextual amplification	Within-country homicide × long-run disinformation	0.24	[−0.45, 0.93]	= 0.495
M3 contextual amplification	Long-run homicide × within-country disinformation	0.52	[0.24, 0.80]	< 0.001
M3 contextual amplification	Within-country homicide × within-country disinformation	0.42	[−0.21, 1.05]	= 0.192
M4 add victimization prevalence	Within-country lagged log homicide	−1.32	[−2.29, −0.36]	= 0.007
M4 add victimization prevalence	Within-country government online disinformation	0.66	[0.13, 1.20]	= 0.016
M4 add victimization prevalence	Within-country homicide × long-run disinformation	0.37	[−0.22, 0.96]	= 0.219
M4 add victimization prevalence	Long-run homicide × within-country disinformation	0.45	[0.19, 0.71]	< 0.001
M4 add victimization prevalence	Within-country homicide × within-country disinformation	0.20	[−0.50, 0.90]	= 0.577
M4 add victimization prevalence	Victimization prevalence	−42.85	[−72.18, −13.53]	= 0.004

*N* = 308 country–round–year cells in 38 countries. Weighted least squares used the number of nonmissing safety responses. Primary models include country and ESS-round fixed effects, log GDP per capita, unemployment, urban population share, and Internet-user share. Standard errors and displayed p values are conventional country-clustered estimates. For Model 3, 9,999-replication restricted wild-cluster bootstrap p values were 0.087 for homicide, 0.052 for disinformation, and 0.091 for the long-run homicide × within-country disinformation interaction. Model 4 additionally controls cell victimization prevalence.

**Figure 2:**
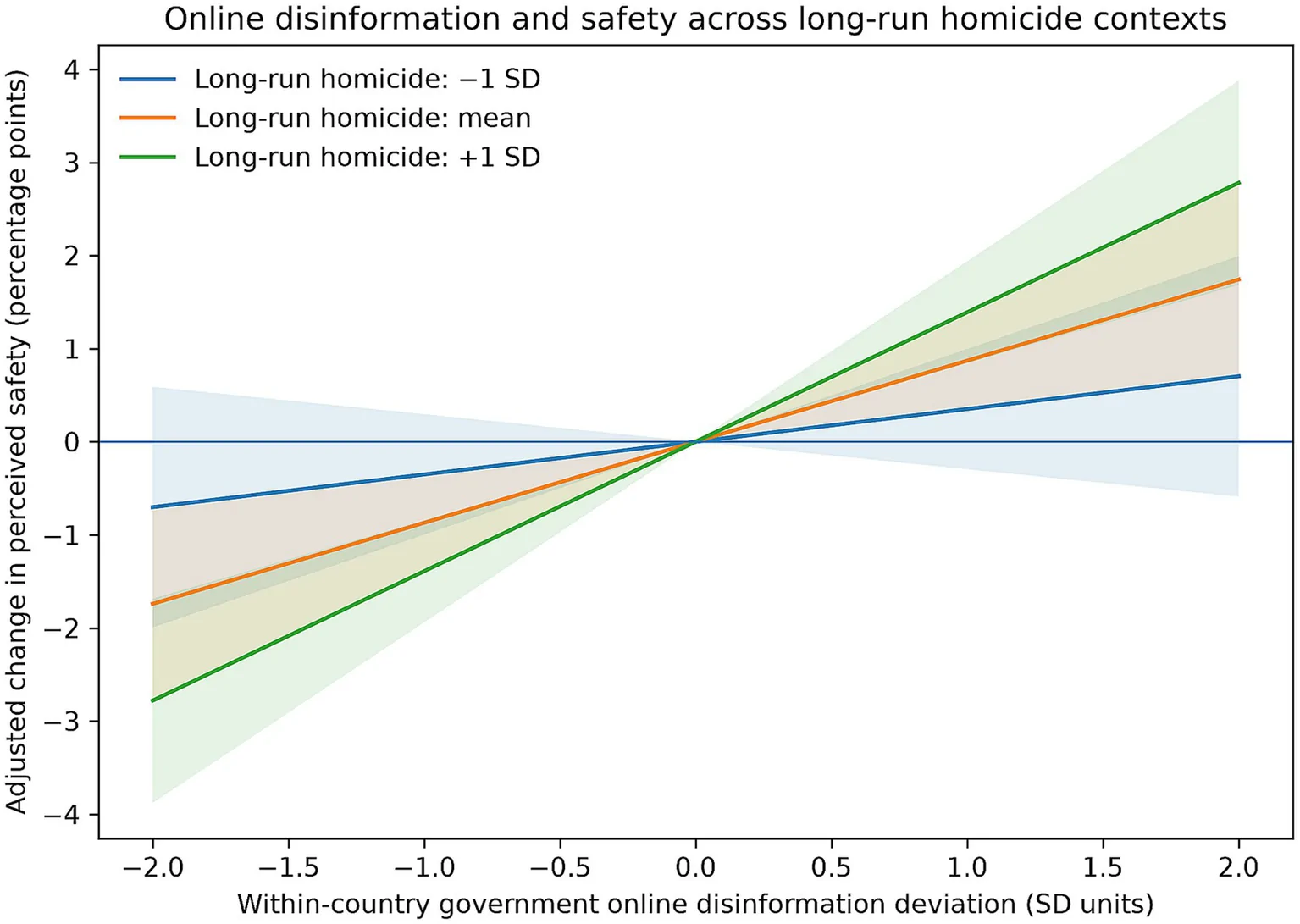
Conditional association between within-country government online disinformation and perceived safety at three levels of long-run homicide. Lines show adjusted predictions from Model 3; shaded bands show 95% confidence intervals. Alt text: Line chart with increasingly positive disinformation slopes as long-run homicide moves from one standard deviation below the mean to one standard deviation above it.

Conventional estimates for RQ1 and RQ2 suggested an attenuation-shaped pattern. A one-SD within-country increase in government online disinformation was associated with a 0.87-point increase in perceived safety (95% CI [0.34, 1.40], *p* = 0.001). The interaction between long-run homicide context and within-country disinformation was +0.52 points (95% CI [0.24, 0.80], *p* < 0.001). The estimated disinformation slope was +0.35 points in countries 1 SD below the mean long-run homicide context (95% CI [−0.29, 1.00]), +0.87 at the mean (95% CI [0.34, 1.40]), and +1.39 at +1 SD (95% CI [0.84, 1.94]) (Figure 2). The other two homicide × disinformation terms were imprecise. These conventional estimates answer the research questions descriptively but require the small-cluster and temporal sensitivity qualifications below.

Model 4 added cell victimization prevalence. The conventional coefficient for victimization prevalence was negative (−42.85 percentage points for a one-unit change, 95% CI [−72.18, −13.53], *p* = 0.004), while the focal coefficient directions remained: homicide −1.32 points (*p* = 0.007), disinformation +0.66 points (*p* = 0.016), and long-run homicide × within-disinformation +0.45 points (*p* < 0.001). This check shows that adjustment for aggregate victimization prevalence did not reverse the conventional coefficient directions; it does not eliminate other time-varying confounding.

**Figure 3:**
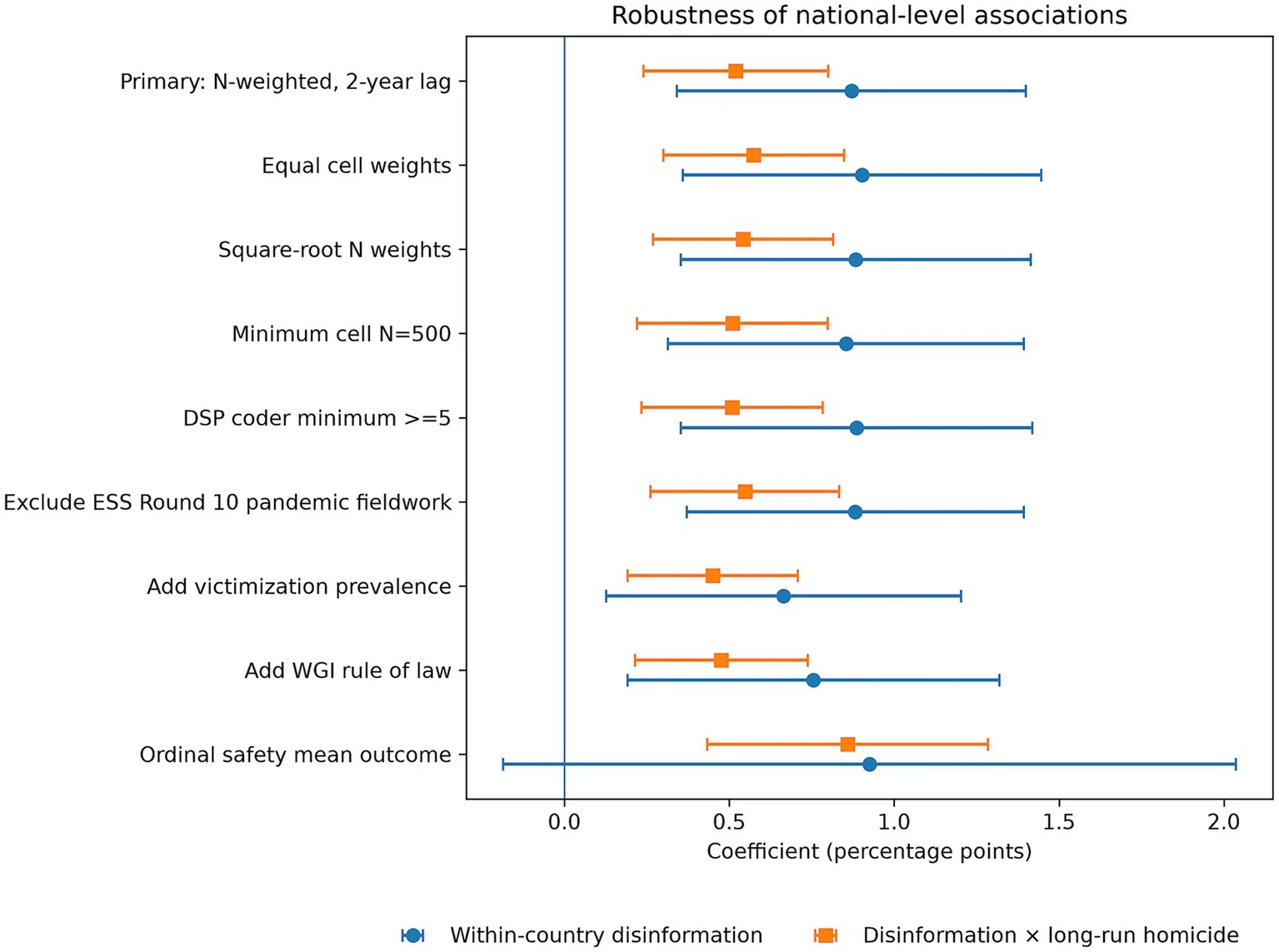
Robustness of national disinformation estimates. Points show coefficients and horizontal bars show 95% confidence intervals across alternative weights, samples, lags, controls, and quality thresholds. Alt text: Forest plot showing generally positive coefficients for within-country government disinformation and its interaction with long-run homicide across specifications.

The focal coefficients were stable when calendar-year fixed effects replaced ESS-round fixed effects: homicide −1.41 points, disinformation +0.81, and the contextual interaction +0.53. Including both round and calendar-year indicators yielded −1.40, +0.79, and +0.53 points, respectively. Country-specific linear trends were more consequential. With round fixed effects and country trends, the homicide and disinformation estimates attenuated to −0.48 (95% CI [−1.71, 0.74], *p* = 0.441) and +0.45 (95% CI [−0.39, 1.29], *p* = 0.294), while the contextual interaction remained positive but imprecise (+0.84, 95% CI [−0.15, 1.83], *p* = 0.098). Calendar-year fixed effects plus country trends produced the same qualification (−0.50, +0.38, and +0.73; all *p* ≥ 0.130). Thus, the national coefficient directions recur across temporal specifications, but their magnitudes and precision are sensitive to country-specific trends. Other coefficient signs were stable under equal-cell and square-root-N weights, a minimum cell size of 500, a five-coder DSP threshold, exclusion of Round 10, alternative one- and three-year lag windows, rule-of-law adjustment, and party-disinformation substitution (Figure 3; Supplementary Tables S7, S8). Leave-one-country-out estimates were positive for disinformation and the contextual interaction in every omission. The ordinal safety outcome reproduced the negative homicide and positive contextual-interaction directions, although the disinformation main effect was less precise. These checks identify directional recurrence, not immunity to temporal confounding.

To make the pooled longitudinal patterns concrete, *post hoc* descriptive within-country contrasts showed that Estonia in 2009 and Lithuania in 2011 combined unusually high lagged homicide with safety 11.4 and 15.6 percentage points below their respective country means. By 2023, when lagged homicide was markedly lower, safety in the same countries stood 12.4 and 12.2 points above their country means. The largest upward deviations in expert-coded government online disinformation occurred in Russia (2017), Hungary (2017–2020), and Poland (2018–2023); several of these cells also had above-country-mean safety. These examples identify high-deviation country–years, not country-specific statistically significant effects, and they contextualize rather than identify the pooled coefficients. The sequence spans the post-2008 recession, later shifts in political communication, and the COVID-19 era, so sociohistorical conditions remain plausible time-varying confounders (Table 3).

**Table 3:** Individual internet use, victimization, and perceived safety.

Model	Term	b (pp)	95% CI	*p*	*N*
I1 internet use	Internet use (within-cell SD)	1.72	[1.26, 2.17]	<0.001	408,149
I1 internet use	Victimization	−13.25	[−15.94, −10.56]	<0.001	408,149
I2 internet × victimization	Internet use (within-cell SD)	1.31	[0.83, 1.78]	<0.001	408,149
I2 internet × victimization	Victimization	−13.65	[−16.36, −10.94]	<0.001	408,149
I2 internet × victimization	Internet use × victimization	2.67	[2.00, 3.34]	<0.001	408,149
I3 internet × government disinformation	Internet use (within-cell SD)	1.36	[0.88, 1.83]	<0.001	408,149
I3 internet × government disinformation	Victimization	−13.67	[−16.38, −10.96]	<0.001	408,149
I3 internet × government disinformation	Internet use × victimization	2.61	[1.96, 3.27]	<0.001	408,149
I3 internet × government disinformation	Internet use × government disinformation	−0.45	[−0.90, −0.01]	= 0.045	408,149
I4 internet × homicide	Internet use (within-cell SD)	1.39	[0.95, 1.84]	<0.001	345,550
I4 internet × homicide	Victimization	−13.90	[−16.80, −11.00]	<0.001	345,550
I4 internet × homicide	Internet use × victimization	2.62	[1.97, 3.27]	<0.001	345,550
I4 internet × homicide	Internet use × lagged homicide	−0.32	[−0.64, −1.33e-03]	= 0.049	345,550

Weighted linear probability models absorb country–round–year cells and cluster standard errors by country. Primary weights are normalized to a mean of 1 within cells, so larger respondent samples contribute more total weight; an equal-total-cell-weight sensitivity analysis is reported in Section 3.3. All models control female indicator, age and age squared, education and missingness, and unemployment. Internet use is standardized within the cell. For Model I2, 9,999-replication restricted wild-cluster bootstrap p values were < 0.001 for Internet use, victimization, and their interaction. I3 and I4 cross-level interactions are exploratory.

The limited number of national clusters nevertheless limits certainty. With 9,999 replications, restricted wild-cluster bootstrap *p*-values for Model 3 were 0.087 for homicide, 0.052 for disinformation, and 0.091 for the contextual interaction. None cleared the conventional 0.05 threshold. Combined with the attenuation under country-specific trends, these results require Study 1 to be interpreted as provisional evidence consistent with objective-risk calibration and information-environment attenuation, not as confirmation of either national association or an underlying mechanism.

### 3.3 Study 2: internet use, victimization, and perceived safety

H2 and H3 received consistent support under both country-clustered and restricted wild-cluster inference. In Model I1, a one-SD increase in within-cell Internet-use frequency was associated with a 1.72-percentage-point higher probability of feeling safe (*p* < 0.001). Under conventional country-clustered inference, Model I2 indicated an Internet-use slope of +1.31 points among nonvictims (95% CI [0.83, 1.78], *p* < 0.001), a − 13.65-point difference associated with victimization at mean Internet use (95% CI [−16.36, −10.94], *p* < 0.001), and a + 2.67-point Internet use × victimization interaction (95% CI [2.00, 3.34], *p* < 0.001). The implied Internet-use slope was +3.98 points among victims, compared with +1.31 points among nonvictims. Restricted wild-cluster bootstrap *p* values were also < 0.001 for Internet use, victimization, and their interaction.

**Figure 4:**
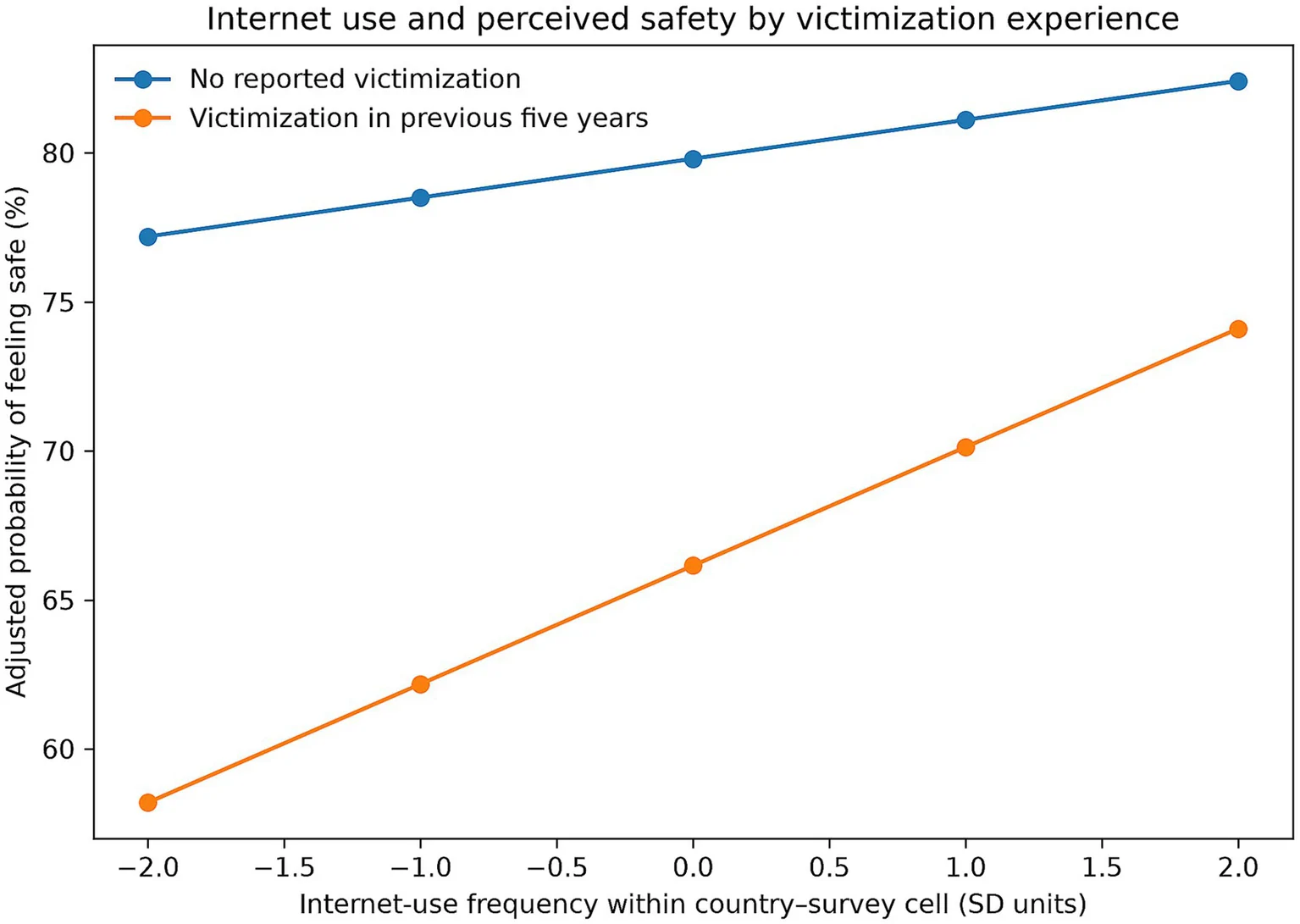
Adjusted probability of feeling safe by within-cell internet-use frequency and victimization. Predictions average over observed covariates and country–round–year contexts. Alt text: Two upward-sloping lines. Respondents reporting victimization start at lower predicted safety but have a steeper positive internet-use slope.

Standardized predictions illustrate the interaction (Figure 4). At mean Internet use, predicted safety was 79.81% for nonvictims and 66.15% for victims. At −2 SD, predictions were 77.20 and 58.20%; at +2 SD they were 82.42 and 74.11%, respectively. Internet use did not eliminate the victimization penalty, but the estimated safety gap narrowed from approximately 19.0 points at low use to 8.3 points at high use. The pattern therefore reflects both persistent vulnerability and differential association with digital embeddedness.

The focal directions also persisted when every country–round–year cell received equal total weight. In that specification, the Internet-use coefficient was +0.94 points (95% CI [0.40, 1.47], *p* < 0.001), victimization was −12.83 points (95% CI [−15.17, −10.50], *p* < 0.001), and the interaction was +2.82 points (95% CI [1.80, 3.84], *p* < 0.001). Additional checks used the ordinal outcome, unweighted estimation, clustering by country–round–year cell, exclusion of Round 10, and household-income adjustment. The pattern appeared in both measurement eras: Rounds 1–5 yielded an Internet coefficient of +1.31 and interaction of +2.00, whereas Rounds 8–11 yielded +1.52 and +3.99 (Supplementary Table S12; Supplementary Figure S2). Income adjustment reduced the main coefficient to +0.89 but did not reverse it; the interaction was +3.47.

Under conventional country-clustered inference, the exploratory cross-level models yielded a 0.45-point reduction in the individual Internet-use slope per SD of national government disinformation (*p* = 0.045) and a 0.32-point reduction per SD of lagged homicide (*p* = 0.049). These estimates were small, were not preregistered, and were not among the focal coefficients evaluated using restricted wild-cluster bootstrap inference. They are reported for transparency as hypothesis-generating patterns and should not be interpreted as confirmatory evidence.

### 3.4 Integrated evidence

Taken together, the studies distinguish processes that a single-level analysis would conflate. Population safety perceptions tracked changes in objective violence within countries. A political digital-information indicator showed a separate contextual association whose statistical certainty depended on the small-cluster correction. Within the same country–round–year context, frequent Internet users—especially those with victimization experience—reported greater safety. The national disinformation measure and individual Internet-use measure are neither interchangeable nor direct measures of crime-information exposure. Their joint value lies in triangulating material risk, information-system context, digital behavior, and lived vulnerability while preserving the limits of each measure.

## 4 Discussion

### 4.1 Principal findings

This study examined perceived safety as a situated psychological judgment patterned by material risk, mediated context, and personal experience. The evidence does not support a universal claim that digital media either amplify fear or produce reassurance. Three qualified patterns emerged. First, conventional models linked within-country homicide increases to lower population safety, but small-cluster inference and country-specific trends made this result provisional. Second, conventional fixed-effects models yielded a counterintuitive positive disinformation coefficient, especially in higher-homicide contexts, but this national pattern was likewise inferentially fragile. Third, respondents who used the Internet more frequently than others in the same national survey context reported greater safety, particularly when they reported victimization; this individual pattern persisted under equal-cell weighting and wild-cluster inference. None of these associations directly measures the psychological mechanisms proposed to interpret it.

### 4.2 Objective-risk calibration and the meaning of perceived safety

The conventional homicide estimate is consistent with a limited form of objective-risk calibration, but it does not establish that process. A detectable negative coefficient under round and calendar-year fixed effects is substantively informative; its attenuation under country-specific trends shows that broad time-varying national trajectories may also account for part of the association.

The magnitude should not be exaggerated. A one-SD rise in lagged log homicide corresponded to about a 1.4-point decline in the primary model, whereas safety varied by more than 10 points across country–round–year cells. National homicide is a rare-event, macro-level sentinel indicator; the ESS item concerns an immediate local setting and may be influenced by nonlethal violence, harassment, burglary, lighting, disorder, and social cohesion. The geographic mismatch, small-cluster uncertainty, and country-trend sensitivity mean that the coefficient is best read as a tentative population-level signal, not evidence that citizens accurately estimate neighborhood risk or that homicide independently causes perceived unsafety.

The finding also extends recent European work. Recent cross-national studies have documented strong roles for individual vulnerability and institutional context, but objective-crime coefficients remain sensitive to whether stable differences and temporal changes are separated (Ejrnæs and Scherg, 2022; Nazzari et al., 2026). The within-between design shows that the question “Are high-crime countries less safe-feeling?” is not equivalent to “Does a country feel less safe when its own homicide level rises?” The latter provides a stricter temporal comparison and reduces confounding by persistent national reporting practices, response styles, and media systems.

### 4.3 Interpreting the contextual disinformation pattern

The positive disinformation coefficient is the most counterintuitive result and the one most vulnerable to overinterpretation. It must not be described as a beneficial effect of false information. DSP v2smgovdom measures expert assessments of government use of social media for misleading domestic communication. It does not identify whether public-safety issues were discussed, whether respondents encountered the content, whether they believed it, or whether the content preceded their neighborhood judgment. The analysis therefore detects a country-level co-movement between an information-system practice and average safety, not a psychological treatment effect.

Risk attenuation remains one possible interpretation, not a measured mechanism. The social amplification framework allows institutional communication to reduce perceived salience through normalization, selective framing, or attention displacement (Kasperson et al., 1988; Kasperson et al., 2022). Research on the disinformation order and informational autocracy describes strategies that manage visibility, attack alternative sources, and maintain favorable performance impressions (Bennett and Livingston, 2018; Guriev and Treisman, 2019). These ideas could explain why a positive contextual coefficient appeared in higher-homicide settings, but the study contains no evidence about message content, exposure, belief, or attention and therefore cannot select this account over competing explanations.

Competing explanations remain credible. The coefficient may reflect unmeasured political cycles, changes in policing or welfare provision, reporting quality, conflict intensity, trust in government, broader regime adaptation, or reverse temporal ordering. Expert coding error may also be correlated across adjacent years. Rule-of-law adjustment, coder restrictions, alternative lag windows, and party-disinformation substitution preserved the signs, but country-specific trends attenuated the main effects and wild-cluster *p* values remained above 0.05. The responsible conclusion is therefore that the data show a directionally recurring but inferentially fragile contextual pattern worthy of direct testing, not a reproducible psychological attenuation mechanism.

A direct test would require information unavailable in ESS: crime-related message content, reach, source attribution, recall, belief, and timing. Content analysis could distinguish reassurance, concealment, blame shifting, and threat inflation. Individual surveys could then measure exposure and perceived credibility. Without those links, the national coefficient remains a contextual signal. This distinction is especially important for media psychology, where moving from a media-system indicator to an individual cognitive mechanism requires evidence about actual encounters and processing.

### 4.4 General internet use as a conditional resource

The individual Internet-use findings were more consistent across the requested checks and more directly comparable with prior survey research. They extend the positive association reported by Ding et al. (2026) across two decades, multiple ESS rounds, 37 countries, and two Internet-frequency instruments. Country–round–year fixed effects compare respondents with different relative use frequencies in the same national-period context. The coefficient direction persisted under equal-cell weighting and household-income adjustment, and the Internet-use, victimization, and interaction terms remained *p* < 0.001 under wild-cluster inference. These checks strengthen confidence in the observed conditional associations but do not resolve selection, reverse ordering, or unmeasured socioeconomic differences.

The result does not contradict evidence that crime-specific digital content can heighten fear. Social-media crime posts disproportionately represent violent events and may track public fear better than actual crime (Prieto Curiel et al., 2020). Social-media consumption and police-scanner group participation have been associated with greater fear in specific samples (Intravia et al., 2017; Kim et al., 2026). The ESS measure, by contrast, is general Internet-use frequency. It includes information seeking but also communication with family and friends, navigation, transport, work, entertainment, service use, and access to emergency or local resources. The distinction between content-specific exposure and general digital embeddedness is therefore central: a person can be highly connected without consuming a high volume of sensational crime content.

Several nonexclusive mechanisms could generate a positive association, but none was directly measured. Digital access may reduce information insufficiency by making local conditions and services easier to check; seeking is associated with information insufficiency, social norms, and personal relevance (Liu et al., 2022). Connectivity may also facilitate communication and access to help, while digital self-efficacy concerns confidence in accomplishing tasks across digital domains (Ulfert-Blank and Schmidt, 2022). Digital inclusion additionally depends on devices, skills, technical support, and usable content (Sieck et al., 2021), and online support-seeking can relate to social capital and perceived community resilience (Xie et al., 2022). These literatures motivate hypotheses linking access and skills to credible information, social support, response efficacy, and perceived control; Internet-use frequency alone does not observe or validate that sequence.

Selection and residual socioeconomic confounding remain equally plausible. People who are healthier, more socially active, wealthier, more mobile, more digital skilled, or more confident may both use the Internet more and feel safer. Reverse ordering is also possible: perceived safety, mobility, and participation may shape Internet use. Income adjustment reduced the main coefficient, indicating that measured socioeconomic resources explain part of the association, while incomplete income coverage and the absence of health, digital-skills, purpose-of-use, support, efficacy, and mobility measures leave substantial residual uncertainty. The study therefore identifies a recurring adjusted association, not informational empowerment as a mediator.

### 4.5 Victimization as differential susceptibility

Victimization was the strongest individual correlate: at mean Internet use, respondents reporting burglary or assault involving themselves or their household were about 13.7 percentage points less likely to feel safe. This aligns with recent longitudinal evidences that direct experience can alter safety and fear, with heterogeneity across victimization histories and neighborhood conditions (Ejrnæs and Scherg, 2022; Kaiser et al., 2025). The interaction is a second descriptive pattern: the positive Internet-use slope was roughly three times larger among victims than nonvictims, and the estimated safety gap narrowed at high use. Equal-cell weighting and wild-cluster inference preserved this pattern, but they do not identify why it occurs.

This interaction is compatible with a person-specific media-effects perspective better than a homogeneous media-effect model (Valkenburg et al., 2021), but it does not directly test differential susceptibility or coping. After victimization, rapid contact, practical information, navigation, peer support, victim services, or monitoring may become more valuable, and risk-information seeking may increase when risk is personally relevant (Liu et al., 2022). Conversely, heightened vigilance could increase exposure to alarming material. The positive adjusted interaction is consistent with several supportive or efficacy-related accounts, but cannot distinguish among them or rule out differential selection.

The alternative possibility is differential selection. Victims who remain highly connected may differ from victims with low Internet use in health, income, social integration, coping resources, or willingness to re-enter public space. The five-year victimization window also means that Internet use may have changed after the event, and the item includes household experiences that may affect respondents indirectly. Longitudinal data are needed to establish whether digital use buffers the psychological aftermath of victimization, whether safer and more resilient victims stay online, or whether both processes operate.

### 4.6 Cross-level integration and contribution to media psychology

The national and individual coefficients describe different constructs rather than one demonstrated process. Government online disinformation is an institutional communication indicator, whereas Internet-use frequency is an individual behavioral measure. A system-level information pattern could coexist with individual access to information and social connection, but the present data do not show that national communication attenuated risk or that individual connectivity increased control. The analytical contribution is to separate levels and formulate testable mechanisms, not to infer those mechanisms from coefficients alone.

This distinction advances media psychology in four respects. First, it embeds media-related variables in an objective-risk model rather than treating perceived safety as an isolated attitude. Second, it separates system-level information quality from individual-level use, reducing the ecological slippage that occurs when Internet penetration is interpreted as personal exposure. Third, it tests direct experience as a theoretically specified susceptibility condition. Fourth, it answers the methodological limitations identified by a recent Frontiers study by extending ESS evidence across 2002–2023 and integrating objective homicide. The resulting account is conditional: media-related associations depend on the level of analysis, informational function, objective environment, and personal vulnerability.

The findings also clarify what triangulation can and cannot accomplish. Agreement across data sources can show that perceived safety is statistically patterned by material conditions, national information indicators, individual Internet use, and victimization. It cannot substitute for direct measurement of exposure, cognition, support, efficacy, or control. The contextual coefficient, Internet-use coefficient, and victimization interaction should therefore be treated as components of a provisional multilevel framework rather than estimates of a causal chain.

### 4.7 Practical implications

Practical implications should remain proportionate to the observational evidence. Public-safety agencies and local governments may consider timely, localized, and verifiable information, clear source attribution, geographic specificity, correction histories, accessible emergency contacts, and links to victim support. These features were not evaluated in the present study and should be tested rather than presented as consequences of the observed Internet-use coefficient. For people with victimization experience, future evaluations could examine whether affordable access, digital-literacy support, verified local information, and human assistance improve information sufficiency, self-efficacy, perceived control, social support, risk calibration, or help-seeking (Sieck et al., 2021).

Digital inclusion alone is insufficient. Access without skills, trusted sources, or usable local information can expose citizens to rumor and emotionally vivid incidents without increasing control. Conversely, reassurance associated with selective visibility or misleading official narratives could coexist with reduced risk recognition and accountability. The provisional national association cannot justify manipulative communication, and the individual association cannot by itself justify a particular intervention. A normatively desirable information environment should instead be evaluated against calibrated safety and effective protective capacity.

### 4.8 Limitations

Several limitations define the boundary of inference. First, both studies use observational repeated cross-sections. Country and country–round–year fixed effects remove stable contextual differences and shared period shocks, but they do not eliminate time-varying confounding, reverse causation, selective Internet use, migration, or policy endogeneity. National estimates were stable when calendar-year fixed effects replaced or supplemented round effects, but the two main effects attenuated under country-specific trends. This sensitivity is substantively important and prevents strong claims about national temporal associations. Lagged exposures establish annual ordering but not causal precedence for the psychological processes proposed. No analysis was preregistered, and the cross-level interactions were exploratory.

Second, the outcome is a single item. It does not distinguish emotional fear, perceived probability, worry frequency, avoidance, or perceived coping capacity, and its hypothetical wording may be interpreted differently by people who do not walk alone after dark. Third, national homicide is geographically distant from the neighborhood setting and omits nonlethal violence, burglary, harassment, incivilities, lighting, and social cohesion. Homicide reporting is more comparable than many police-recorded offenses, but it is not measurement-error free. Fourth, the victimization item combines respondent and household burglary or assault over 5 years, preventing offense-specific and event-time analysis.

Fifth, Internet-use frequency identifies neither platform nor content, purpose, device, digital skill, algorithmic exposure, or active versus passive use. Harmonization across the old and new ESS items preserves relative frequency but cannot make the instruments identical. Sixth, DSP coding is national, political, and expert based. It does not measure crime-related communication, individual reach, belief, or message effects. Seventh, 38 national clusters constrain Study 1 inference, and its wild-cluster results remain above 0.05. Study 2 also has only 37 country clusters; wild-cluster tests supported its three focal terms, but the large respondent count does not create additional independent national clusters and the exploratory cross-level *p* values remain uncertain. Eighth, ESS fieldwork modes and conditions changed over two decades, including the COVID-19 period. Period effects, exclusion checks, and country trends probe these changes but cannot remove all mode or composition differences.

Finally, the design lacks the proposed mediators. We cannot test whether Internet use operates through efficacy, local knowledge, social support, service access, or mobility, nor whether national disinformation operates through attention displacement, favorable framing, source distrust, or concealment. The linear probability models provide interpretable adjusted differences, and ordinal robustness analyses assess recurrence under alternative specifications. Neither converts associations into mechanisms, eliminates selection and socioeconomic confounding, or makes exploratory results confirmatory.

### 4.9 Future research

Future research should connect geocoded panel surveys to local police records, victimization surveys, built-environment measures, and time-stamped communication data. Experience-sampling designs could separate immediate situational safety from generalized beliefs. Passive or donated digital traces, combined with content coding, could distinguish exposure to official alerts, interpersonal support, crime news, rumor, and sensational video. Surveys should measure digital skills, source credibility, efficacy, information insufficiency, social support, and protective behavior so that competing mechanisms can be tested rather than inferred.

Stronger causal designs are also feasible. Broadband expansion, platform outages, changes in emergency-alert systems, local-news closures, or abrupt communication-policy reforms may provide quasi-experimental variation. Panel studies following victims before and after an incident could assess whether digital resources buffer recovery or simply identify resilient users. At the national level, researchers should test whether information-environment changes predict not only perceived safety but also accuracy relative to local risk, protective behavior, trust, reporting, and subsequent victimization. Those outcomes would distinguish adaptive reassurance from maladaptive attenuation.

## 5 Conclusion

Perceived safety across Europe was statistically patterned by objective violence, national digital context, individual connectivity, and victimization, but the strength of evidence differed by level. Conventional national models yielded a negative homicide coefficient and a positive government-disinformation coefficient that was larger in higher-homicide contexts. Calendar-year specifications preserved their directions, whereas country-specific trends attenuated the main effects and restricted wild-cluster *p* values remained above 0.05; these national results are therefore suggestive and unsuitable for causal or normative claims. At the individual level, frequent Internet use was associated with higher safety within country–round–year cells, and the association was stronger among respondents reporting victimization. Equal-cell weighting preserved the directions, and wild-cluster tests supported the three focal individual terms. These associations are compatible with a multilevel media-psychological framework, but they do not demonstrate digital empowerment, reassurance, information access, social support, efficacy, or perceived control. Direct measures and stronger causal designs are required to establish those mechanisms.

## Data Availability

The datasets presented in this study can be found in online repositories. The names of the repository/repositories and accession number(s) can be found at: https://doi.org/10.5281/zenodo.20834833.

## References

[ref1] Andersson D. (2025). News media use and crime perceptions: the dual role of ideology. Mass Commun. Soc. 28, 511–529. doi: 10.1080/15205436.2025.2471866, 40265041 PMC12011023

[ref2] Bell A. Jones K. (2015). Explaining fixed effects: random effects modeling of time-series cross-sectional and panel data. Polit. Sci. Res. Methods 3, 133–153. doi: 10.1017/psrm.2014.7

[ref3] Bennett W. L. Livingston S. (2018). The disinformation order: disruptive communication and the decline of democratic institutions. Eur. J. Commun. 33, 122–139. doi: 10.1177/0267323118760317

[ref4] Camacho Doyle M. Gerell M. Andershed H. (2022). Perceived unsafety and fear of crime: the role of violent and property crime, neighborhood characteristics, and prior perceived unsafety and fear of crime. Deviant Behav. 43, 1347–1365. doi: 10.1080/01639625.2021.1982657

[ref5] Digital Society Project (2026). Digital Society Survey, Version 8. Available online at: https://digitalsocietyproject.org/data/ (Accessed June 23, 2026).

[ref6] Ding Z. Li Z. Luo Y. Wang Y. Liu Z. (2026). Digital technology use intensity and public safety perception: the moderating roles of victimization experience and safety values. Front. Psychol. 17:1836160. doi: 10.3389/fpsyg.2026.1836160, 42317683 PMC13272415

[ref7] Ecker U. K. H. Lewandowsky S. Cook J. Schmid P. Fazio L. K. Brashier N. . (2022). The psychological drivers of misinformation belief and its resistance to correction. Nat. Rev. Psychol. 1, 13–29. doi: 10.1038/s44159-021-00006-y

[ref8] Ejrnæs A. Scherg R. H. (2022). The impact of victimization on feelings of unsafety in different welfare regimes. Eur. J. Criminol. 19, 1304–1326. doi: 10.1177/1477370820960025

[ref9] European Social Survey European Research Infrastructure (2024). European Social Survey, Rounds 1–11. Available online at: https://ess.sikt.no/ (Accessed June 20, 2026).

[ref10] Freelon D. Wells C. (2020). Disinformation as political communication. Polit. Commun. 37, 145–156. doi: 10.1080/10584609.2020.1723755

[ref11] Guriev S. Treisman D. (2019). Informational autocrats. J. Econ. Perspect. 33, 100–127. doi: 10.1257/jep.33.4.100

[ref12] Hart T. C. Chataway M. Mellberg J. (2022). Measuring fear of crime during the past 25 years: a systematic quantitative literature review. J. Crim. Justice 82:101988. doi: 10.1016/j.jcrimjus.2022.101988

[ref13] Humprecht E. Esser F. Van Aelst P. (2020). Resilience to online disinformation: a framework for cross-national comparative research. Int. J. Press/Polit. 25, 493–516. doi: 10.1177/1940161219900126

[ref14] Intravia J. Wolff K. T. Paez R. Gibbs B. R. (2017). Investigating the relationship between social media consumption and fear of crime. Comput. Hum. Behav. 77, 158–168. doi: 10.1016/j.chb.2017.08.047

[ref15] Kaiser F. Oberwittler D. Janssen H. J. Hoffman L. (2025). Exploring heterogeneous effects of victimization on changes in fear of crime: the moderating role of neighborhood conditions. J. Justice Q. 42, 1–30. doi: 10.1080/07418825.2024.2304775

[ref16] Kasperson R. E. Renn O. Slovic P. Brown H. S. Emel J. Goble R. . (1988). The social amplification of risk: a conceptual framework. Risk Anal. 8, 177–187. doi: 10.1111/j.1539-6924.1988.tb01168.x

[ref17] Kasperson R. E. Webler T. Ram B. Sutton J. (2022). The social amplification of risk framework: new perspectives. Risk Anal. 42, 1367–1380. doi: 10.1111/risa.13926, 35861634 PMC10360138

[ref18] Kim D. Lee H. D. Armocida M. (2026). Live-crime update: are police scanner social media users more fearful of violent crime? Int. J. Police Sci. Manag. doi: 10.1177/14613557261419283

[ref19] Liu Z. Yang J. Z. Feeley T. H. (2022). Reduced RISK information seeking model (RISK): a meta-analysis. Sci. Commun. 44, 787–813. doi: 10.1177/10755470221144453

[ref20] MacKinnon J. G. Nielsen M. Ø. Webb M. D. (2023). Cluster-robust inference: a guide to empirical practice. J. Econom. 232, 272–299. doi: 10.1016/j.jeconom.2022.04.001

[ref21] Näsi M. Tanskanen M. Kivivuori J. Haara P. Reunanen E. (2021). Crime news consumption and fear of violence: the role of traditional media, social media, and alternative information sources. Crime Delinq. 67, 574–600. doi: 10.1177/0011128720922539

[ref22] Nazzari M. Triventi M. Calaresu M. (2026). Fear of crime in context: a cross-national longitudinal analysis of perceived unsafety in Europe, 2002–2020. Glob. Crime 27, 1–28. doi: 10.1080/17440572.2025.2539853

[ref23] Prieto Curiel R. Cresci S. Muntean C. I. Bishop S. R. (2020). Crime and its fear in social media. Palgrave Commun. 6:57. doi: 10.1057/s41599-020-0430-7

[ref24] Rosenberger J. S. Dierenfeldt R. Ingle H. (2023). Media consumption and fear of crime: evidence of the need for an intersectional approach. Victims Off. 18, 691–714. doi: 10.1080/15564886.2021.1991069

[ref25] Shin S. Y. Watson B. R. (2024). Trial by media? Media use, fear of crime, and attitudes toward police. Journal. Pract. 18, 1015–1036. doi: 10.1080/17512786.2022.2055624, 37339054

[ref26] Sieck C. J. Sheon A. Ancker J. S. Castek J. Callahan B. Siefer A. (2021). Digital inclusion as a social determinant of health. npj Digit. Med. 4:52. doi: 10.1038/s41746-021-00413-8, 33731887 PMC7969595

[ref27] Spielvogel I. Koban K. Matthes J. (2024). How news consumption in modern media landscapes relates to threat perceptions and fears in public spaces: a scoping review. J. Risk Res. 27, 458–480. doi: 10.1080/13669877.2024.2350717

[ref28] Ulfert-Blank A.-S. Schmidt I. (2022). Assessing digital self-efficacy: review and scale development. Comput. Educ. 191:104626. doi: 10.1016/j.compedu.2022.104626

[ref29] Valkenburg P. M. Beyens I. Pouwels J. L. van Driel I. I. Keijsers L. (2021). Social media use and adolescents’ self-esteem: heading for a person-specific media effects paradigm. J. Commun. 71, 56–78. doi: 10.1093/joc/jqaa039

[ref9001] Visser M. Scholte M. Scheepers P. (2013). Fear of crime and feelings of unsafety in European countries: macro and micro explanations in cross-national perspective. Soc. Q. 54, 278–301. doi: 10.1111/tsq.1202

[ref30] Wang Z. Liu H. Zhou L. Zhang W. Zhou M. (2022). Does internet use affect citizens’ perception of social safety? A cross-sectional survey in China. Systems 10:232. doi: 10.3390/systems10060232

[ref31] World Bank (2026). World Development Indicators. Available online at: https://data.worldbank.org/ (Accessed June 6, 2026).

[ref32] Xie L. Pinto J. Zhong B. (2022). Building community resilience on social media to help recover from the COVID-19 pandemic. Comput. Hum. Behav. 134:107294. doi: 10.1016/j.chb.2022.107294, 35431426 PMC8994552

