# Supplementary material for: Perceived safety in digital information environments: objective violence, online disinformation, internet use, and victimization across Europe, 2002–2023
Source: Front Psychol. 2026 Aug 26;17:1918767. doi: 10.3389/fpsyg.2026.1918767 (PMC13553534; doi:10.3389/fpsyg.2026.1918767)
Supplement: Supplementary file 1 [file Table_1.docx]

Supplementary Material

Supplementary material for: Perceived safety in digital information environments: Objective violence, government online disinformation, Internet use, and victimization across Europe, 2002–2023

# 1 S1. Data sources and analytic structure

The analysis combined ESS Rounds 1–11, Digital Society Project v8, World Development Indicators, UNODC homicide data distributed through WDI, and Worldwide Governance Indicators for one robustness model. Macro exposures were aligned to the interview year and averaged over t−1 and t−2. Country–round–year cells were the national-panel unit. The individual study retained respondent records from rounds with harmonizable Internet-frequency measures and absorbed country–round–year cells.

# 2 S2. Sample flow

**Supplementary Table S1. Analytic sample flow.**

| **Analysis** | **Stage** | **N / represented N** | **Countries** | **Cells** |
| --- | --- | --- | --- | --- |
| ESS integrated respondent file | All respondents, Rounds 1–11 | 518,597 | 39 | 433 |
| National panel | Complete safety, homicide, disinformation, and controls; cell N>=200; DSP coders>=4 | 442,100 | 38 | 308 |
| Individual Internet analysis | Rounds with harmonizable Internet-use measure and complete core variables | 408,149 | 37 | 345 |

**Supplementary Table S2. Country coverage.**

| **Country** | **National cells** | **Individual cells** | **National represented N** |
| --- | --- | --- | --- |
| ALB | 2 | 0 | 1,201 |
| AUT | 8 | 7 | 15,400 |
| BEL | 1 | 16 | 1,787 |
| BGR | 7 | 7 | 15,352 |
| CHE | 9 | 16 | 10,961 |
| CYP | 7 | 6 | 5,846 |
| CZE | 10 | 9 | 16,373 |
| DEU | 19 | 13 | 28,120 |
| DNK | 4 | 11 | 6,050 |
| ESP | 13 | 13 | 18,845 |
| EST | 9 | 13 | 13,890 |
| FIN | 15 | 12 | 20,923 |
| FRA | 17 | 12 | 20,719 |
| GBR | 11 | 16 | 16,169 |
| GRC | 5 | 6 | 12,909 |
| HRV | 6 | 8 | 7,941 |
| HUN | 11 | 10 | 18,487 |
| IRL | 16 | 15 | 23,891 |
| ISL | 6 | 7 | 4,006 |
| ISR | 10 | 8 | 15,655 |
| ITA | 6 | 6 | 12,935 |
| LTU | 7 | 5 | 13,017 |
| LUX | 2 | 3 | 3,141 |
| LVA | 2 | 4 | 2,087 |
| MKD | 1 | 1 | 1,429 |
| MNE | 3 | 3 | 4,087 |
| NLD | 18 | 16 | 19,834 |
| NOR | 11 | 14 | 15,187 |
| POL | 9 | 13 | 11,964 |
| PRT | 9 | 16 | 10,257 |
| ROU | 2 | 2 | 2,146 |
| RUS | 6 | 7 | 12,376 |
| SRB | 2 | 3 | 3,423 |
| SVK | 12 | 10 | 12,734 |
| SVN | 11 | 13 | 14,442 |
| SWE | 14 | 14 | 16,734 |
| TUR | 3 | 4 | 4,117 |
| UKR | 4 | 6 | 7,665 |

# 3 S3. Variable coding and harmonization

**Supplementary Table S3. Core measures.**

| **Construct** | **Source variable** | **Operationalization** |
| --- | --- | --- |
| Perceived safety | ESS aesfdrk | Binary: very safe/safe = 1; unsafe/very unsafe = 0. Ordinal robustness: 1–4, higher safer. |
| Victimization | ESS crmvct | Respondent or household burglary/assault in prior five years; yes = 1. |
| Internet use, R1–R5 | ESS netuse | Rescaled from 0–7 to 0–1, then standardized within country–round–year. |
| Internet use, R8–R11 | ESS netusoft | Rescaled from 1–5 to 0–1, then standardized within country–round–year. |
| Objective violence | WDI VC.IHR.PSRC.P5 / UNODC | log[1 + mean homicide rate per 100,000 in t−1 and t−2]. |
| Government online disinformation | DSP v2smgovdom | Reversed so higher = more disinformation; mean t−1 and t−2. |
| Party disinformation | DSP v2smpardom | Reversed so higher = more party disinformation; alternative specification. |
| Online pluralism | DSP v2smonper | Mean t−1 and t−2; exploratory because DSP flags measurement-model convergence caution. |

# 4 S4. National-panel descriptives and models

**Supplementary Table S4. National-panel descriptives.**

| **Variable** | **N** | **Mean** | **SD** | **Min** | **Max** |
| --- | --- | --- | --- | --- | --- |
| Proportion feeling safe | 308 | 0.788 | 0.104 | 0.410 | 0.952 |
| Ordinal safety | 308 | 3.024 | 0.258 | 2.342 | 3.594 |
| Victimization prevalence | 308 | 0.162 | 0.069 | 0.026 | 0.344 |
| Homicide rate, lag mean | 308 | 1.757 | 2.422 | 0.480 | 25.842 |
| log(1 + homicide) | 308 | 0.871 | 0.440 | 0.392 | 3.290 |
| Government disinformation | 308 | -1.770 | 0.975 | -2.957 | 2.383 |
| Party disinformation | 308 | -1.476 | 1.019 | -3.202 | 2.028 |
| Online pluralism | 308 | 1.442 | 0.566 | -1.205 | 2.642 |
| Online fragmentation | 308 | -0.545 | 1.018 | -2.630 | 2.322 |
| Internet users (%) | 308 | 72.918 | 20.406 | 3.750 | 99.533 |
| Real GDP per capita | 308 | 33914.376 | 20549.115 | 2122.602 | 100292.567 |
| Unemployment (%) | 308 | 7.796 | 3.775 | 2.246 | 26.094 |
| Urban population (%) | 308 | 72.929 | 10.933 | 49.762 | 95.322 |

**Supplementary Table S5. Focal coefficients from national models.**

| **Model** | **Term** | **b (pp)** | **SE** | **95% CI** | **p** | **N** |
| --- | --- | --- | --- | --- | --- | --- |
| M1 objective risk | log_hom_wz | -1.614 | 0.595 | [-2.781, -0.448] | = .007 | 308 |
| M2 add disinformation | log_hom_wz | -1.638 | 0.598 | [-2.810, -0.466] | = .006 | 308 |
| M2 add disinformation | disinfo_wz | 0.801 | 0.429 | [-0.041, 1.642] | = .062 | 308 |
| M3 contextual amplification | log_hom_wz | -1.424 | 0.534 | [-2.471, -0.377] | = .008 | 308 |
| M3 contextual amplification | disinfo_wz | 0.871 | 0.270 | [0.342, 1.400] | = .001 | 308 |
| M3 contextual amplification | h_w_x_dis_b | 0.241 | 0.353 | [-0.451, 0.932] | = .495 | 308 |
| M3 contextual amplification | h_b_x_dis_w | 0.519 | 0.143 | [0.239, 0.799] | < .001 | 308 |
| M3 contextual amplification | h_w_x_dis_w | 0.421 | 0.323 | [-0.212, 1.054] | = .192 | 308 |
| M4 add victimization prevalence | log_hom_wz | -1.324 | 0.494 | [-2.292, -0.356] | = .007 | 308 |
| M4 add victimization prevalence | disinfo_wz | 0.665 | 0.275 | [0.126, 1.203] | = .016 | 308 |
| M4 add victimization prevalence | h_w_x_dis_b | 0.368 | 0.300 | [-0.219, 0.956] | = .219 | 308 |
| M4 add victimization prevalence | h_b_x_dis_w | 0.450 | 0.132 | [0.191, 0.708] | < .001 | 308 |
| M4 add victimization prevalence | h_w_x_dis_w | 0.199 | 0.357 | [-0.501, 0.900] | = .577 | 308 |
| M4 add victimization prevalence | victim_share | -42.853 | 14.961 | [-72.177, -13.529] | = .004 | 308 |

**Supplementary Table S6. Simple slopes and wild-cluster bootstrap inference.**

| **Analysis** | **Quantity** | **Estimate (pp)** | **95% CI** | **Inference** |
| --- | --- | --- | --- | --- |
| Simple slope | Disinformation at homicide -1 SD | 0.351 | [-0.294, 0.997] | Cluster-robust CI |
| Simple slope | Disinformation at homicide +0 SD | 0.871 | [0.342, 1.400] | Cluster-robust CI |
| Simple slope | Disinformation at homicide +1 SD | 1.390 | [0.842, 1.937] | Cluster-robust CI |
| Wild bootstrap | log_hom_wz | — | — | p = 0.095; 999 reps |
| Wild bootstrap | disinfo_wz | — | — | p = 0.053; 999 reps |
| Wild bootstrap | h_b_x_dis_w | — | — | p = 0.085; 999 reps |

# 5 S5. National-panel robustness

**Supplementary Table S7. Selected robustness specifications.**

| **Specification** | **Term** | **b (pp)** | **95% CI** | **p** | **Cells** |
| --- | --- | --- | --- | --- | --- |
| Primary: N-weighted, 2-year lag | log_hom_wz | -1.424 | [-2.471, -0.377] | = .008 | 308 |
| Primary: N-weighted, 2-year lag | disinfo_wz | 0.871 | [0.342, 1.400] | = .001 | 308 |
| Primary: N-weighted, 2-year lag | h_b_x_dis_w | 0.519 | [0.239, 0.799] | < .001 | 308 |
| Equal cell weights | log_hom_wz | -1.424 | [-2.434, -0.415] | = .006 | 308 |
| Equal cell weights | disinfo_wz | 0.903 | [0.360, 1.446] | = .001 | 308 |
| Equal cell weights | h_b_x_dis_w | 0.574 | [0.300, 0.848] | < .001 | 308 |
| Square-root N weights | log_hom_wz | -1.416 | [-2.442, -0.389] | = .007 | 308 |
| Square-root N weights | disinfo_wz | 0.884 | [0.353, 1.414] | = .001 | 308 |
| Square-root N weights | h_b_x_dis_w | 0.542 | [0.268, 0.815] | < .001 | 308 |
| Minimum cell N=500 | log_hom_wz | -1.406 | [-2.479, -0.332] | = .010 | 267 |
| Minimum cell N=500 | disinfo_wz | 0.854 | [0.315, 1.393] | = .002 | 267 |
| Minimum cell N=500 | h_b_x_dis_w | 0.510 | [0.221, 0.799] | < .001 | 267 |
| DSP coder minimum >=5 | log_hom_wz | -1.515 | [-2.546, -0.484] | = .004 | 300 |
| DSP coder minimum >=5 | disinfo_wz | 0.886 | [0.354, 1.419] | = .001 | 300 |
| DSP coder minimum >=5 | h_b_x_dis_w | 0.508 | [0.233, 0.784] | < .001 | 300 |
| Add victimization prevalence | log_hom_wz | -1.324 | [-2.292, -0.356] | = .007 | 308 |
| Add victimization prevalence | disinfo_wz | 0.665 | [0.126, 1.203] | = .016 | 308 |
| Add victimization prevalence | h_b_x_dis_w | 0.450 | [0.191, 0.708] | < .001 | 308 |
| Add WGI rule of law | log_hom_wz | -1.251 | [-2.300, -0.201] | = .020 | 292 |
| Add WGI rule of law | disinfo_wz | 0.755 | [0.192, 1.319] | = .009 | 292 |
| Add WGI rule of law | h_b_x_dis_w | 0.476 | [0.214, 0.738] | < .001 | 292 |
| Party disinformation alternative | log_hom_wz | -0.835 | [-1.722, 0.051] | = .065 | 308 |
| Party disinformation alternative | party_wz | 1.345 | [0.299, 2.391] | = .012 | 308 |
| Party disinformation alternative | h_b_x_party_w | 0.626 | [0.280, 0.973] | < .001 | 308 |
| Party disinformation alternative | h_w_x_party_b | 0.729 | [0.099, 1.359] | = .023 | 308 |

**Supplementary Table S8. Leave-one-country-out coefficient ranges.**

| **Term** | **Omissions** | **Minimum** | **Maximum** | **Mean** | **Summary** |
| --- | --- | --- | --- | --- | --- |
| disinfo_wz | 38 | 0.631 | 1.104 | 0.877 | All estimates retained the primary positive sign |
| h_b_x_dis_w | 38 | 0.458 | 1.265 | 0.540 | All estimates retained the primary positive sign |

# 6 S6. Individual-study descriptives and models

**Supplementary Table S9. Individual-study weighted descriptives.**

| **Variable** | **N** | **Weighted mean** | **Weighted SD** | **Min** | **Max** |
| --- | --- | --- | --- | --- | --- |
| safe_binary | 408,149 | 0.777 | 0.416 | 0.000 | 1.000 |
| safe_ord_higher_safer | 408,149 | 3.009 | 0.792 | 1.000 | 4.000 |
| internet01 | 408,149 | 0.616 | 0.431 | 0.000 | 1.000 |
| internet_z | 408,149 | -8.91e-18 | 1.000 | -7.612 | 4.422 |
| victimization | 408,149 | 0.161 | 0.368 | 0.000 | 1.000 |
| female | 408,149 | 0.517 | 0.500 | 0.000 | 1.000 |
| age | 408,149 | 46.415 | 18.653 | 15.000 | 100.000 |
| eduyrs | 403,272 | 12.363 | 4.013 | 0.000 | 40.000 |
| unemployed | 408,149 | 0.043 | 0.202 | 0.000 | 1.000 |

**Supplementary Table S10. Individual focal models.**

| **Model** | **Term** | **b (pp)** | **SE** | **95% CI** | **p** | **N** |
| --- | --- | --- | --- | --- | --- | --- |
| I1 Internet use | internet_z | 1.717 | 0.231 | [1.264, 2.171] | < .001 | 408,149 |
| I1 Internet use | victimization | -13.252 | 1.373 | [-15.944, -10.561] | < .001 | 408,149 |
| I2 Internet × victimization | internet_z | 1.305 | 0.242 | [0.831, 1.780] | < .001 | 408,149 |
| I2 Internet × victimization | victimization | -13.653 | 1.382 | [-16.362, -10.943] | < .001 | 408,149 |
| I2 Internet × victimization | internet_x_victim | 2.671 | 0.342 | [2.002, 3.341] | < .001 | 408,149 |
| I3 Internet × government disinformation | internet_z | 1.358 | 0.243 | [0.882, 1.833] | < .001 | 408,149 |
| I3 Internet × government disinformation | victimization | -13.672 | 1.384 | [-16.385, -10.959] | < .001 | 408,149 |
| I3 Internet × government disinformation | internet_x_victim | 2.613 | 0.334 | [1.958, 3.268] | < .001 | 408,149 |
| I3 Internet × government disinformation | internet_x_gov_disinfo_lag2mean | -0.454 | 0.226 | [-0.898, -0.011] | = .045 | 408,149 |
| I4 Internet × homicide | internet_z | 1.394 | 0.225 | [0.952, 1.836] | < .001 | 345,550 |
| I4 Internet × homicide | victimization | -13.900 | 1.480 | [-16.800, -10.999] | < .001 | 345,550 |
| I4 Internet × homicide | internet_x_victim | 2.622 | 0.333 | [1.969, 3.274] | < .001 | 345,550 |
| I4 Internet × homicide | internet_x_homicide_rate_lag2mean | -0.318 | 0.162 | [-0.635, -0.001] | = .049 | 345,550 |

**Supplementary Table S11. Selected standardized predicted probabilities.**

| **Victimization status** | **Internet use (SD)** | **Predicted safety** |
| --- | --- | --- |
| Nonvictim | -2.0 | 77.20% |
| Nonvictim | 0.0 | 79.81% |
| Nonvictim | 2.0 | 82.42% |
| Victim | -2.0 | 58.20% |
| Victim | 0.0 | 66.15% |
| Victim | 2.0 | 74.11% |

**Supplementary Table S12. Individual-model robustness.**

| **Specification** | **Term** | **Estimate** | **95% CI** | **p** | **N** |
| --- | --- | --- | --- | --- | --- |
| Primary binary, weighted | internet_z | 1.305 | [0.831, 1.780] | < .001 | 408,149 |
| Primary binary, weighted | internet_x_victim | 2.671 | [2.002, 3.341] | < .001 | 408,149 |
| Ordinal outcome | internet_z | 0.026 | [0.016, 0.036] | < .001 | 408,149 |
| Ordinal outcome | internet_x_victim | 0.046 | [0.035, 0.058] | < .001 | 408,149 |
| Old Internet scale, rounds 1–5 | internet_z | 1.310 | [0.759, 1.860] | < .001 | 230,061 |
| Old Internet scale, rounds 1–5 | internet_x_victim | 1.998 | [1.287, 2.710] | < .001 | 230,061 |
| New Internet scale, rounds 8–11 | internet_z | 1.519 | [0.969, 2.068] | < .001 | 178,088 |
| New Internet scale, rounds 8–11 | internet_x_victim | 3.985 | [2.866, 5.104] | < .001 | 178,088 |
| Unweighted | internet_z | 1.392 | [0.948, 1.836] | < .001 | 408,149 |
| Unweighted | internet_x_victim | 2.866 | [2.239, 3.493] | < .001 | 408,149 |
| Cluster by country–round–year | internet_z | 1.305 | [1.015, 1.595] | < .001 | 408,149 |
| Cluster by country–round–year | internet_x_victim | 2.671 | [2.161, 3.181] | < .001 | 408,149 |
| Exclude ESS Round 10 | internet_z | 1.258 | [0.770, 1.745] | < .001 | 371,375 |
| Exclude ESS Round 10 | internet_x_victim | 2.636 | [1.953, 3.320] | < .001 | 371,375 |
| Add household income decile | internet_z | 0.893 | [0.478, 1.307] | < .001 | 222,618 |
| Add household income decile | internet_x_victim | 3.469 | [2.495, 4.443] | < .001 | 222,618 |

**Supplementary Table S13. Individual-study round and fieldwork-year coverage.**

| **ESS round** | **Fieldwork year** | **Respondents** | **Countries** | **Cells** |
| --- | --- | --- | --- | --- |
| 1 | 2002 | 25,925 | 16 | 16 |
| 1 | 2003 | 11,046 | 15 | 15 |
| 2 | 2004 | 27,808 | 17 | 17 |
| 2 | 2005 | 15,104 | 19 | 19 |
| 2 | 2006 | 1,659 | 1 | 1 |
| 3 | 2006 | 31,704 | 22 | 22 |
| 3 | 2007 | 10,167 | 18 | 18 |
| 4 | 2008 | 29,343 | 22 | 22 |
| 4 | 2009 | 25,519 | 28 | 28 |
| 4 | 2010 | 421 | 1 | 1 |
| 5 | 2010 | 22,067 | 19 | 19 |
| 5 | 2011 | 29,156 | 24 | 24 |
| 5 | 2012 | 142 | 1 | 1 |
| 8 | 2016 | 25,944 | 18 | 18 |
| 8 | 2017 | 17,709 | 20 | 20 |
| 9 | 2018 | 26,471 | 21 | 21 |
| 9 | 2019 | 21,648 | 27 | 27 |
| 9 | 2020 | 552 | 4 | 4 |
| 10 | 2020 | 36,774 | 22 | 22 |
| 11 | 2023 | 48,990 | 30 | 30 |

**Supplementary Table S14. Exploratory cross-level moderation.**

| **Model** | **Term** | **b (pp)** | **95% CI** | **p** | **N** |
| --- | --- | --- | --- | --- | --- |
| I3 Internet × government disinformation | internet_x_gov_disinfo_lag2mean | -0.454 | [-0.898, -0.011] | = .045 | 408,149 |
| I4 Internet × homicide | internet_x_homicide_rate_lag2mean | -0.318 | [-0.635, -0.001] | = .049 | 345,550 |

# 7 S7. Supplementary figures

**Supplementary Figure S1. Weighted perceived safety across ESS rounds in the individual analysis sample.**

**Supplementary Figure S2. Internet-use and Internet × victimization associations in the primary sample and the two measurement eras. Points are coefficients; bars are 95% confidence intervals.**

# 8 S8. Data access and licensing

ESS respondent-level data are available after free registration from the ESS Data Portal and are not redistributed in the accompanying package. Digital Society Project and World Bank data are openly downloadable from their official portals. The public replication package contains non-disclosive country–survey-cell aggregates, exact model outputs, figures, analysis code, and a source manifest. The datasets analyzed for this study can be found in the Zenodo, https://doi.org/10.5281/zenodo.20834833.
